# The trends of women’s autonomy in health care decision making and associated factors in Ethiopia: evidence from 2005, 2011 and 2016 DHS data

**DOI:** 10.1186/s12905-021-01517-9

**Published:** 2021-10-26

**Authors:** Melkamu Dires Asabu, Derebe Kelkay Altaseb

**Affiliations:** 1 Department of Political Science and International Relations, Faculty of Social Sciences and Humanities, Woldia University, Woldia, Ethiopia; 2 Department of Statistics, Faculty of Natural and Competition Sciences, Woldia University, Woldia, Ethiopia

**Keywords:** Autonomy, Decision making, Ethiopia, Health care, Women

## Abstract

**Background:**

Women's autonomy in health care decision-making is very crucial for the well-being of women themselves, their children, and the entire family members. Although studying the issue is significant to take proper interventions, the issue is not studied at a nationwide level in Ethiopia. Accordingly, this population-based nationwide study was aimed at assessing the trends of women’s autonomy in health care decision-making and its associated factors in Ethiopia.

**Method:**

The sample was limited to married women of 2005 (n = 8617), 2011 (n = 10,168), and 2016 (n = 9824) Ethiopian Demographic and Health Survey (EDHS) data. Women's autonomy in health care decision-making was measured based on their response to the question ‘person who usually decides on respondent's health care. To examine associated factors, socio-demographic variables were computed using multinomial logistic regression.

**Result:**

The finding revealed that the trend of women’s autonomy in health care decision-making had declined from 18.7% in 2005 to 17.2% in 2011 albeit it had risen to 19.1% in 2016. The autonomy of women who resides in urban areas was 98.7% higher than rural residents, and those who live in the Tigray region, Somali region, and Addis Ababa are 76.6%, 79.7%, and 95.7% higher than who live in Dire Dawa respectively. Unemployed women, women aged from 15 to 24 years, and uneducated women were 45.1%, 32.4%, and 32.2% less likely autonomous in health care decision making respectively.

**Conclusion:**

The autonomy of women in health care decision-making had declined from 2005 to 2011. Therefore, the role of stakeholders in taking possible interventions like empowering women shall be strengthened. This is to protect women from certain health problems as well as for the well-being of women themselves, their children, and the entire family members.

## Introduction

Since women’s participation in the decision-making process is very crucial to achieve the multifaceted development of communities and countries [1], the global community is working to empower women in enhancing their decision-making ability including at the household level. For instance, achieving gender equality and give power to all women and girls are the prime focus among seventeen identified “Sustainable Development Goals” for the transformation and quality of life of the world [2]. To achieve the proposed “Sustainable Development Goals” through taking proper interventions, studying women's autonomy in health care decision-making and its associated factors is compulsory. Furthermore, there is no doubt on the importance of assessing the position of women in decision-making to address the problem of gender inequality via devising policies and taking immediate intervention measures to empower women [3–6]. However, this can be possible when the present position of women’s autonomy including in health care decision making is known.

The word ‘autonomy’ can be defined as a technical, social, and psychological ability of an individual for making decisions about his/her private concerns independently [7]. It also refers that the independence to make one own choice and decision [8]. An individual is autonomous when she/he can act under her/his direction, i.e. to make her/his actions regarding one's owns personal problems [9]. Accordingly, women's autonomy means the capacity and freedom women have to act or decide independently on their issues including on their own health care issues. Women’s autonomy in health care decision-making is very crucial for the well-being of women themselves. This is due to the fact that the ability of women to visit health care facilities and receive treatment is dependent on their ability in making personal decisions [10]. Moreover, women's health care decision-making autonomy is also important in enhancing the well-being of their children and the entire family [11].

Despite the fact that since no culture is free from gender-based discrimination, women are adversely affecting to contribute their share [12]. This exposed them to have a low level of participation in most decision-making [13, 14]. In many cultures across the world, women are denied their right to participate in decision-making and determine their fates. This is especially serious among women who have husbands or marital partners. In this regard, the power inequalities at the household level between husbands and wives can restrict the health decision-making autonomy of women. This limits their utilization of health services which affects reproductive health outcomes and creates problems for their families, communities, and nations at large [15].

With regard to Ethiopian women’s autonomy in health care decision making, there is a single local study that was focused on rural Southern Ethiopian women specifically in Wolayita and Dawro Zones’ rural women. However, due to geographic delimitation and cultural affiliation, it is difficult to generalize local study’s findings at a country or Ethiopian level. For this reason, studying the trends of women’s autonomy in health care decision making at a national level is more necessary to understand the current autonomy of women in health care decision making and devise national policies in accordance with the finding of empirical study. Therefore, since the national level trends of women's autonomy and its associated factors in health care decision making is an overlooked research area in Ethiopia, this study’s objective was focused on examining the trends of women's autonomy in health care decision-making and its associated factors in Ethiopia. Since the study had used 2005, 2011, and 2016 Ethiopian DHS data, the finding will help to understand the trends of women's autonomy from time to time and its associated factors. Above all, the study will strongly inform policy makers and other stakeholders to take immediate intervention measures to empower women in order to improve their autonomy in health care decision making in Ethiopia.

## Objectives of the study

Using 2005, 2011 and 2016 Ethiopian DHS data, this study’s objectives were: to examine the trends of women’s autonomy in health care decision making in Ethiopia; and to identify factors associated with women’s autonomy in health care decision making in Ethiopia.

## Methods

### Study design and data collection

The study used data from 2005, 2011, and 2016 Ethiopian DHS that were collected by the Central Statistical Authority (CSA) of Ethiopia and Opinion Research Corporation Company (ORC) Macro International. It was conducted in all Regional States of Ethiopia namely Tigray, Afar, Amhara, Oromia, Somali, Benishangul Gumuz, Southern Nations Nationalities and Peoples (SNNP), Gambella and Harari as well as in Addis Ababa, and Dire Dawa city Administrations [16]. The data is a nationally representative sample survey, aged 15–49 years’ women.

Although the survey collected information from unmarried, married, living with a partner, divorced and widowed women, in this study attention had been given to women who are married and living with their partners. This is because the power inequalities at the household level between women and husbands/partners may restrict the health decision-making autonomy of women than other women [15]. Based on the valid number of responses for identified variables, the sample size of the study from 2005, 2011, and 2016 DHS data were limited to 8617, 10,168, and 9824 respectively.

### Variables and measurement

#### Dependent variable

The study’s dependent variable was women's autonomy in health care decision-making. This was measured based on women's response to ‘person who usually decides on respondent's health care. The responses of this dependent variable were coded as (1) independently, (2) together with others, and (3) others. This is due to the fact that an individual is autonomous when she/he can act under her/his direction, i.e. to make her/his actions regarding one's owns personal problems [9]. Thus, women who decide independently on their health were considered as they are autonomous in health care decision making.

#### Independent variables

The survey collected a detailed woman's background characteristics. Abroad studies identified women’s age and educational status as well as household wealth index as a determinant factor for women’s health care decision-making autonomy [8]. Moreover, studies also identified working status [15], place of residence [17], religion and region [18]. Therefore, based on their possible impacts upon women's autonomy in health care decision-making, this study identified the following independent variables including women’s age, education status, working status, place of residence, household wealth index, religion, and region. The researchers adopted the measurements of the DHS survey for some selected independent variables, while the measurements of the DHS survey on the following three variables such as age, education level, and household wealth index were adapted as follows.

In the DHS, age of the respondents was open to writing their exact age, but a study that focused on modern contraceptive measured the age of respondents by labeling from aged 15–24, 25–34, and 35–49 [8, 19]. To measure age, this study had used 11–24, 25–34, and 35–49 age categories of women. In the case of educational attainment, the DHS used six responses such as no education, incomplete primary, primary, incomplete secondary, secondary and higher. In this regard, studies that were done using DHS data on "the effect of maternal health service utilization in early initiation of breastfeeding among Nepalese mothers" [20] as well as “women empowerment and their reproductive behavior among currently married women in Ethiopia” [21] had used 'illiterate', 'primary', 'secondary' and 'higher' to measure this variable. Accordingly, incomplete primary and primary, and incomplete secondary and secondary were merged into 'primary' and 'secondary' respectively. About the wealth index, the middle was taken as it is but the categories poorest and poor as well as rich and richest were merged into poor and rich respectively. Comparable to this adapted measurement, other studies [10, 22–24] had used this measurement to measure the wealth index.

### Data analysis

The data obtained from 2005, 2011, and 2016 Ethiopian DHS were analyzed through SPSS version 22 in three levels. First, descriptive statistics were used to summarize the socio-demographic variables of the study participants using frequency and percentages. Finally, analysis of the determinants of women's autonomy in health care decision making was carried out using logistic regression, particularly multinomial logistic regression. This is because logistic regression is used to examine the relationships between a categorical outcome variable and one or more categorical or continuous predictor variables [25]. Principally, multinomial logistic regression is applied in cases where the dependent variable is categorized in three and more nominal responses [26]. This is because the dependent variable (women autonomy in health care decision making) was coded as (1) independently, (2) together with others, and (3) others. To analyze women’s autonomy in health care decision making, using multiple multinomial logistic regressions, the researchers did comparisons of women’s autonomy among “those who pass a decision on their health care issues independently with others” as well as “those who pass a decision on their health care issues independently with together”. For analysis of multiple multinomial logistic regression analyses, statistical inferences were made based on estimates of the odds ratio (OR) with a 95% confidence level and 5% margin of error or *p* value less than .05. As we can observe in Table 1 that had shown model fitting information summary, − 2 log-likelihood of the intercept only model is greater than − 2 log likelihood of the final (saturated) model that gives a conclusion of the final model is better. In addition to this at a 95% level of significance, the *p* value of the likelihood ratio tests of the final model is less than zero which gives the same conclusion as the above. This statistical significance indicates that at least one independent variable had a significant effect on the dependent one. This statistical significance indicates that at least one independent variable had shown a significant effect on the dependent one.

**Table 1:** Multiple multinomial logistic regression model fitting information summary

Model	Model fitting criteria	Likelihood ratio tests		
	− 2 log likelihood	Chi-Square	Df	Sig.
Intercept only	6409.214			
Final	5449.749	959.465	46	.000

## Results

As we have seen in Table 2, there is a significant decrement of women’s autonomy in health care decision-making. Ethiopian women's autonomy in health care decision making whose age is 24 and below had declined from 23.9% in 2005 to 23.1% and 22.3% in 2011 and 2016 respectively. Similarly, the autonomy of women who are aged from 35 to 49 also declined from 38.7% in 2005 to 37.6% and 36.4% in 2011 and 2016 respectively. The autonomy of uneducated women in health care decision-making had also declined from 68.5% in 2005 to 52.5% and 51.6% in 2011 and 2016 respectively. The autonomy of housewife women in health care decision-making had declined from 68.4% in 2005 to 56.1% in 2011. However, the status of employed women in health care decision-making had risen from 31.64% in 2005 to 43.9% in 2011 albeit it fails into 41.8% in 2016. The autonomy of rural women in health care decision-making had declined from 68.9% in 2005 to 60.1% in 2011. Health care decision-making autonomy of women who were from poor household wealth index had declined from 37.5% in 2005 to 32.5% in 2011. Muslim women's autonomy in health care decision-making had declined from 43.6% in 2005 to 39.2% in 2011 but it had risen to 42% in 2016. Among the two Federal Cities Administrations and nine Regional States, the autonomy of women in health care decision making in three Regional States including in Amhara, Oromia, and Harari had shown significant decrements from 2005 to 2011 and 2016.

**Table 2:** Description of socio-demographic variables of women

Background characteristics of women		Women autonomy in health care decision making								
		2005, n = 8617			2011, n = 10,108			2016, n = 9824		
		Self	Together	Others	Self	Together	Others	Self	Together	Others
Age	< 24	385 (23.9%)	1052 (25.4%)	784 (27.3%)	405 (23.1%)	1397 (24.3%)	762 (28.5%)	419 (22.3%)	1517 (24.8%)	511 (27.9%)
	25–34	602 (37.4%)	1649 (39.8%)	1168 (40.7%)	688 (39.2%)	2445 (42.6%)	1051 (39.3%)	774 (41.2%)	2595 (42.4%)	721 (39.4%)
	35–49	622 (38.7%)	1440 (34.8%)	915 (31.9%)	660 (37.6%)	1898 (33.1%)	862 (32.2%)	684 (36.4%)	2004 (32.8%)	599 (32.7%)
Educational status	No education	1102 (68.5%)	2925 (70.6%)	2345 (81.8%)	921 (52.5%)	3637 (63.4%)	1987 (74.3%)	968 (51.6%)	3473 (56.8%)	1252 (68.4%)
	Primary	237 (14.7%)	663 (16.0%)	415 (14.5%)	531 (30.3%)	1589 (27.7%)	610 (22.8%)	548 (29.2%)	1710 (28.0%)	442 (24.1%)
	Secondary	213 (13.2%)	475 (11.5%)	100 (3.5%)	174 (9.9%)	298 (5.2%)	54 (2.0%)	218 (11.6%)	572 (9.4%)	90 (4.9%)
	Higher	57 (3.5%)	78 (1.9%)	7 (.2%)	127 (7.2%)	216 (3.8%)	24 (.9%)	143 (7.6%)	361 (5.9%)	47 (2.6%)
Working status	Housewife	1100 (68.4%)	3031 (73.2%)	2331 (81.3%)	983 (56.1%)	3887 (67.7%)	1988 (74.3%)	1092 (58.2%)	4167 (68.1%)	1377 (75.2%
	Employed	509 (31.6%)	1110 (26.8%)	536 (18.7%)	770 (43.9%)	1853 (32.3%)	687 (25.7%)	785 (41.8%)	1949 (31.9%)	454 (24.8%)
Residence	Urban	500 (31.1%)	920 (22.2%)	278 (9.7%)	699 (39.9%)	1378 (24.0%)	337 (12.6%)	656 (34.9%)	1596 (26.1%)	239 (13.1%)
	Rural	1109 (68.9%)	3221 (77.8%)	2589 (90.3%)	1054 (60.1%)	4362 (76.0%)	2338 (87.4%)	1221 (65.1%)	4520 (73.9%)	1592 (86.9%)
Wealth Index	Poor	604 (37.5%)	1522 (36.8%)	1420 (49.5%)	570 (32.5%)	2359 (41.1%)	1459 (54.5%)	795 (42.4%)	2513 (41.1%)	1098 (60.0%)
	Middle	186 (11.6%)	693 (16.7%)	560 (19.5%)	208 (11.9%)	916 (16.0%)	454 (17.0%)	229 (12.2%)	888 (14.5%)	242 (13.2%)
	Rich	819 (50.9%)	1926 (46.5%)	887 (30.9%)	975 (55.6%)	2465 (42.9%)	762 (28.5%)	853 (45.4%)	2715 (44.4%)	491 (26.8%)
Religion	Orthodox	642 (39.9%)	2171 (52.4%)	888 (31.0%)	706 (40.3%)	2397 (41.8%)	631 (23.6%)	719 (38.3%)	2329 (38.1%)	487 (26.6%)
	Catholic	14 (.9%)	33 (.8%)	33 (1.2%)	26 (1.5%)	48 (.8%)	37 (1.4%)	12 (.6%)	38 (.6%)	10 (.5%)
	Protestant	212 (13.2%)	604 (14.6%)	599 (20.9%)	308 (17.6%)	994 (17.3%)	579 (21.6%)	329 (17.5%)	1083 (17.7%)	372 (20.3%)
	Muslim	702 (43.6%)	1243 (30.0%)	1263 (44.1%)	687 (39.2%)	2241 (39.0%)	1348 (50.4%)	788 (42.0%)	2614 (42.7%)	916 (50.0%)
	Others	39 (2.4%)	90 (2.2%)	84 (2.9%)	26 (1.5%)	60 (1.0%)	80 (3.0%)	29 (1.5%)	52 (.9%)	46 (2.5%)
Region	Tigray	78 (4.8%)	440 (10.6%)	277 (9.7%)	223 (11.9%)	588 (9.6%)	146 (8.0%)	223 (11.9%)	588 (9.6%)	146 (8.0%)
	Afar	114 (7.1%)	286 (6.9%)	214 (7.5%)	149 (7.9%)	467 (7.6%)	250 (13.7%)	149 (7.9%)	467 (7.6%)	250 (13.7%)
	Amhara	153 (9.5%)	848 (20.5%)	293 (10.2%)	132 (7.0%)	850 (13.9%)	146 (8.0%)	132 (7.0%)	850 (13.9%)	146 (8.0%)
	Oromia	168 (10.4%)	760 (18.4%)	539 (18.8%)	149 (7.9%)	913 (14.9%)	255 (13.9%)	149 (7.9%)	913 (14.9%)	255 (13.9%)
	Somali	176 (10.9%)	87 (2.1%)	243 (8.5%)	289 (15.4%)	452 (7.4%)	237 (12.9%)	289 (15.4%)	452 (7.4%)	237 (12.9%)
	Benishangul Gumuz	69 (4.3%)	320 (7.7%)	241 (8.4%)	118 (6.3%)	521 (8.5%)	167 (9.1%)	118 (6.3%)	521 (8.5%)	167 (9.1%)
	SNNP	249 (15.5%)	562 (13.6%)	551 (19.2%)	225 (12.0%)	724 (11.8%)	268 (14.6%)	225 (12.0%)	724 (11.8%)	268 (14.6%)
	Gambela	101 (6.3%)	186 (4.5%)	219 (7.6%)	198 (10.5%)	337 (5.5%)	177 (9.7%)	198 (10.5%)	337 (5.5%)	268 (14.6%)
	Harari	234 (14.5%)	121 (2.9%)	129 (4.5%)	56 (3.0%)	473 (7.7%)	47 (2.6%)	56 (3.0%)	473 (7.7%)	47 (2.6%)
	Addis Ababa	174 (10.8%)	314 (7.6%)	53 (1.8%)	230 (12.3%)	397 (6.5%)	50 (2.7%)	230 (12.3%)	397 (6.5%)	50 (2.7%)
	Dire Dawa	93 (5.8%)	217 (5.2%)	108 (3.8%)	108 (5.8%)	394 (6.4%)	88 (4.8%)	108 (5.8%)	394 (6.4%)	88 (4.8%)

**Fig. 1:**
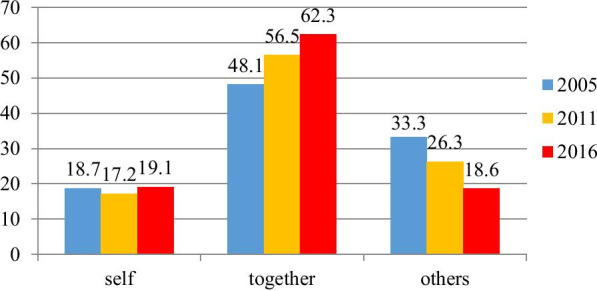
Women’s autonomy in health care decision making

As it had been seen in Fig. 1, women's autonomy in health care decision-making had declined from 18.7% in 2005 to 17.2% in 2011 albeit it had rose into 19.1% in 2016.

It is obvious that a multiple multinomial logistic regression used to examine the effect of all independent variables on the dependent one at a time. The multiple multinomial logistic regression output is interpreted as the effect of one independent variable on the dependent one at a constant value of another covariate or factor independents. As we have seen below, Table 3 gives information about multivariable multinomial logistic regression analysis output of this study.

**Table 3:** Multivariable multinomial logistic regression analysis of associated factors on women’s autonomy in health care decision making (adjusted odds ratio)

	Self with others		Self with together	
	COR (95% CI)	AOR (95% CI)	COR (95% CI)	AOR (95% CI)
*Residence*				
Urban	3.579 (3.032, 4.224)***	1.987 (1.553, 2.543)***	1.522 (1.362, 1.700)***	1.366 (1.138, 1.640)***
Rural^®^	1	1	1	1
*Region*				
Tigray	1.245 (.877, 1.767)	1.766 (1.177, 2.649)**	1.384 (1.064, 1.799)*	1.538** (1.141, 2.073)
Afar	.486 (.343, .687)***	.974 (.673, 1.408)	1.164 (.878, 1.543)	1.422* (1.056, 1.915)
Amhara	.737 (.510, 1.063)	1.172 (.776, 1.770)	.567 (.428, .750)***	.685 (.501, .935)*
Oromia	.476 (.337, .673)***	.748 (.517, 1.084)	.595 (.452, .783)***	.703 (.526, .941)*
Somali	.994 (.715, 1.382)	1.797 (1.267, 2.550)**	2.333 (1.800, 3.022)***	2.742 (2.084, 3.608)***
Benishangul	.576 (.399, .831)**	.791 (.534, 1.172)	.826 (.617, 1.106)***	.848 (.622, 1.156)
SNNP	.684 (.491, .954)*	1.074 (.730, 1.580)	1.134 (.874, 1.471)	1.425 (1.056, 1.923)*
Gambela	.911 (.644, 1.289)	1.216 (.818, 1.808)	2.143 (1.627, 2.824)***	2.500 (1.836, 3.403)***
Harari	.971 (.601, 1.568)	1.082 (.665, 1.762)	.432 (.305, .613)***	.454 (.319, .646)***
Addis Ababa	3.748 (2.473, 5.680)***	1.957 (1.250, 3.062)**	2.114 (1.617, 2.762)***	1.855 (1.394, 2.467)***
Dire Dawa^®^	1	1	1	1
*Education level*				
No education	.254 (.181, .357)***	.678 (.459, 1.000)*	.704 (.572, .865)***	.951 (.740, 1.221)
Primary	.407 (.286, .580)***	.996 (681, 1.457)	.809 (.652, 1.004)	1.193 (.940, 1.515)
Secondary	.796 (.528, 1.201)	1.320 (.863, 2.019)	.962 (.750, 1.234)	1.147 (.884, 1.488)
Higher^®^	1	1	1	1
*Religion*				
Orthodox	2.342 (1.451, 3.780)***	1.182 (.706, 1.978)	.554 (.349, .879)*	.551 (.338, .900)*
Catholic	1.903 (.729, 4.968)	1.371 (.514, 3.660)	.566 (.256, 1.250)	.503 (.223, 1.133)
Protestant	1.403 (.861, 2.285)	1.004 (.607, 1.663)	.545 (.340, .872)*	.450 (.277, .732)***
Muslim	1.365 (.849, 2.193)	1.167 (.702, 1.941)	.541 (.341, .857)**	.571 (.350, .929)*
Other^®^	1	1	1	1
*Wealth index*				
Poor	.417 (.361, .481)***	.877 (.718, 1.072)	.007 (.901, 1.125)*	1.262 (1.071, 1.486)**
Middle	.545 (.441, .673)***	1.139 (.891, 1.455)	.821 (.696, .967)*	1.305 (1.071, 1.590)**
Rich^®^	1	1	1	1
*Work status*				
Housewife	.459 (.399, .528)***	.549 (.472, .639)***	.651 (.585, .724)***	.649 (.577, .729)***
Employed^®^	1	1	1	1
*Age category*				
15–24 years	.718 (.606, .851)***	.676 (.561, .816)***	.809 (.704, .930)**	.762 (.654, .889)***
25–34 years	.940 (.810, 1.092)	.892 (.764, 1.041)	.874 (.776, .984)*	.840 (.742, .950)**
35–49 years^®^	1	1	1	1

^*®*^Reference category, *COR* crude odds ratio, *AOR* adjusted odds ratio, *CI* confidence interval

***p value < .001, **p value < .01,*p value < .05

This section deals with women’s autonomy in comparison with those who passed decisions independently and women who passed decisions with others together on their health care issues. In this regard, the autonomy of women in health care decision making among those who live in urban areas was more likely higher than women who live in rural areas (AOR = 1.366; 95% CI = 1.138, 1.640). The levels of women’s autonomy who resides in Tigray (AOR = 1.538; 95% CI = 1.141, 2.073), Afar (AOR = 1.422; 95% CI = 1.056, 1.915), SNNP (AOR = 1.425; 95% CI = 1.056, 1.923), Gambela (AOR = 2.500; 95% CI = 1.836, 3.403), Somali (AOR = 2.742; 95% CI = 2.084, 3.608) and Addis Ababa (AOR = 1.855; 95% CI = 1.394, 2.467) were more likely higher in comparison with those who live in Dire Dawa city administration. Inversely, women who reside in Benishangul (AOR = .848; 95% CI = .622, 1.156), Harari (AOR = .454; 95% CI = .319, .646), Amhara AOR = .685; 95% CI = .501, .935) and Oromia (AOR = .703; 95% CI = .526, .941) were less likely autonomous than those who live in Dire Dawa city administration. Based on religions what women follow, women from Orthodox Christianity (AOR = .551; 95% CI = .338, .900), Protestant (AOR = .450; 95% CI = .277, .732), and Islamic (AOR = .571; 95% CI = .350, .929) religions were less likely autonomous than those who were from other religions. Women who were from poor and middle wealth index households were 1.262 times (AOR = 1.262; 95% CI = 1.071, 1.486) and 1.305 times (AOR = 1.305; 95% CI = 1.071, 1.590) more likely autonomous than those who were from rich wealth index households, respectively. Housewife women were less likely autonomous when compared with those who were employed women (AOR = .649; 95% CI = .577, .729). Women from 15–24 (AOR = .762; 95% CI = .654, .889) to 25–34 (AOR = .840; 95% CI = .742, .950) years old were less likely autonomous than those who were from 35 to 49 years old.

In this section, women’s autonomy was analyzed based on the comparison of women who passed decisions independently and women who didn’t pass a decision about their health care issue. The autonomy of women who were from urban areas was more likely higher in comparison with those who were from rural areas (AOR = 1.987; 95% CI = 1.553, 2.543). The levels of women’s autonomy who reside in Addis Ababa city administration, Tigray, and Somali regional states were 1.797 times, 1.766 times, and 1.797 times more likely higher than women who reside in Dire Dawa city administration, respectively. In comparison with employed women, housewife women were less likely autonomous than those who were employed women (AOR = .549; 95% CI = .472, .639). Concerning women’s age, women aged from 15 to 24 years were less likely autonomous than those were aged from 35 to 49 years (AOR = .676; 95% CI = .561, .816).

## Discussion

Women's autonomy in health care decision-making had declined from 18.7% in 2005 to 17.2% in 2011 though it also had risen to 19.1% in 2016. In the same way, the other study that focused on the autonomy of women in health care decision making revealed a declination trend of women’s autonomy in refusing risky sex [27]. This declination of women’s autonomy might be associated with the enactment of restrictive legislation of the country in 2009 that potentially diminish the role of civil society organizations in promoting democratic values [28].

In regard to associated factors of women’s autonomy in health care decision making, the study’s finding revealed that the autonomy of women who resides in urban areas was 98.7% more likely higher than rural residents. Similarly, the other studies conducted in Bangladesh as well as in Nigeria found higher mothers’ autonomy of urban women than rural women [17, 18, 29, 30]. The possible justification might be associated with the fact that urban women have better exposer to mass media. Because among women those who are exposed to mass media are more likely active in decision making [31].

The autonomy of women who live in Tigray, Somali and Addis Ababa was more likely higher in health care decision making than those who live in Dire Dawa city administration respectively. Similar to this study finding, other related studies [8, 15, 18, 27] found a significant difference of women's autonomy across different geographic regional states. This might be because of the contributions and commitments of regional governments in empowering women may vary from one to another.

Similar to other researches findings’ [15, 18, 29, 30, 32] and feminists’ assumption [33–35], the autonomy of employed women in health care decision making was more likely higher than housewife or unemployed women. Moreover, in consistency with many other related studies [8, 17, 18, 29, 30], women with poor household were less like autonomous in health care decision making. The reason might be that the economic dependence of women makes them to develop too low self-confidence to engage in decision making because ‘low self-esteem associated with low economic status’ [36].

The autonomy of women who were aged 15–24 years was found a lower position (by 32.4%) than women from 35 to 49 years of age. Corresponding to this study’s finding, others studies which focused on women’s autonomy in health care decision making [8, 10, 17, 18, 29], household decision making [15], refusing risky sex [27] and on reproductive human rights [37] the age of respondents had significant effect. This might be due to the fact that “older age is associated with decreases in self-esteem” [38].

Women who had no formal education were by 32.2% less likely autonomous than those who attain higher education. Consistently, the finding of other studies [8, 17, 18, 29, 30, 39, 40] revealed a positive association of women's autonomy with their educational status. This might be due to the fact that “highly educated women are more likely to be knowledgeable about their rights and health, have more self-confidence and be more assertive than those with less or no education” [8].

## Conclusion and recommendation

Women's autonomy in health care decision-making is very crucial for the well-being of women themselves, their children, and the entire family members. This study was aimed at assessing the trends of women's autonomy in health care decision-making and its associated factors in Ethiopia. Accordingly, the finding revealed that the trend of women's autonomy in health care decision-making had shown a declination trend. The autonomy of women who resides in urban areas was higher than rural residents. With regard to geographic areas women who reside in Benishangul, Harari, Amhara, and Oromia regional states were less likely autonomous than those who live in Dire Dawa city administration. In terms of religion, women from Orthodox Christianity, Protestant, and Islamic religions were less likely autonomous than those who were from other religions. Housewife women were less likely autonomous when compared with those who were employed women from 15–24 to 25–34 years old were less likely autonomous than those who were from 35 to 49 years old. Therefore, the role of stakeholders in taking possible intervention measures like empowering women shall be strengthened. This is especially for women who from rural parts of the country, women from Benishangul, Harari, Amhara, and Oromia regional states, women from Orthodox Christianity, Protestant, and Islamic religion followers, unemployed or housewife women, as well as women aged from 15–24 to 25–34 years old needs special attention of stakeholders. Accordingly, both government organizations and non-governmental bodies shall provide proper training that can boost women's self-confidence as well as women’s understanding about their rights in accessing and actively participating in health care decision making.

### The strengths and limitations of the study

The study contains both strengths and limitations. One of the major strengths of the study is that it revealed the autonomy of women in health care decision making and its associated factors in Ethiopia or at a national level. It is the first nationwide study that may help to devise policies and to women empowerment specifically in health care decision making at a country level. Despite these strengths, the study has the following limitations. The first limitation is associated with self-reported data and the limitations that the multivariable model may have missed some variables that could potentially have been relevant. Moreover, since the DHS data were collected in a cross-sectional design, the study can establish associations but not establish causality. The other limitation is that since the study used a collected data from the Ethiopian DHS, it lacks triangulation of data using diverse methods of data collection.

## Data Availability

The study used a released survey datasets that is available without participants’ identities. This was accessed based on a publicly available dataset that is freely available at http://dhsprogram.com/data/dataset/Ethiopia_Standard-DHS_2016.cfm?fag=0.
